# Biostatistics: essential concepts for the clinician

**DOI:** 10.1590/2177-6709.26.1.E21SPE1

**Published:** 2021-03-10

**Authors:** Darlyane TORRES, David NORMANDO

**Affiliations:** 1 Universidade Federal do Pará, Programa de Pós-Graduação em Odontologia (Belém/PA, Brazil); 2 Universidade Federal do Pará, Departamento de Odontologia (Belém/PA, Brazil)

**Keywords:** Biostatistics, Statistical analysis, Data presentation, Interactive tutorial

## Abstract

**Introduction:**

The efficiency of clinical procedures is based on practical and theoretical knowledge. Countless daily information is available to the orthodontist, but it is up to this professional to know how to select what really has an impact on clinical practice. Evidence-based orthodontics ends up requiring the clinician to know the basics of biostatistics to understand the results of scientific publications. Such concepts are also important for researchers, for correct data planning and analysis.

**Objective:**

This article aims to present, in a clear way, some essential concepts of biostatistics that assist the clinical orthodontist in understanding scientific research, for an evidence-based clinical practice. In addition, an updated version of the tutorial to assist in choosing the appropriate statistical test will be presented. This PowerPoint^®^ tool can be used to assist the user in finding answers to common questions about biostatistics, such as the most appropriate statistical test for comparing groups, choosing graphs, performing correlations and regressions, analyzing casual, random or systematic errors.

**Conclusion:**

Researchers and clinicians must acquire or recall essential concepts to understand and apply an appropriate statistical analysis. It is important that journal readers and reviewers can identify when statistical analyzes are being inappropriately used.

## RESUMO

**Introdução:**

A eficiência dos procedimentos clínicos é baseada em conhecimentos práticos e teóricos. Inúmeras informações diárias estão ao alcance do ortodontista; porém cabe a esse profissional saber selecionar o que realmente tem impacto na prática clínica. A Ortodontia baseada em evidências acaba exigindo que o clínico conheça os fundamentos da bioestatística para compreender os resultados das publicações científicas. Tais conceitos também são importantes aos pesquisadores para um correto planejamento e análise dos dados.

**Objetivo:**

O presente artigo tem como objetivo apresentar, de forma clara, alguns conceitos essenciais da bioestatística que auxiliem o ortodontista clínico na compreensão da pesquisa científica para uma prática clínica baseada em evidências. Além disso, será apresentada uma versão atualizada do tutorial para auxílio na escolha do teste estatístico adequado. Essa ferramenta em PowerPoint® pode ser empregada para auxiliar o usuário a encontrar respostas para dúvidas comuns sobre bioestatística, como o teste estatístico mais adequado para comparar grupos, escolha de gráficos, realizar correlações e regressões, análises de sobrevivência e dos erros aleatório e sistemático.

**Conclusão:**

Pesquisadores e clínicos devem adquirir ou relembrar conceitos essenciais para compreender e aplicar uma análise estatística apropriada. É importante que os leitores e revisores de periódicos possam identificar quando análises estatísticas estão sendo utilizadas de forma inadequada.

## INTRODUCTION

Every professional, regardless of the area of training, has a role in decision-making based on theoretical and practical knowledge. Regarding health professionals, where it is essential to maintain or promote the health of the patient, any inappropriate decision may cause irreversible biological damage to patients. Currently, Orthodontics has been submitted to an avalanche of new information, technologies and experiences, which are easily accessible. And it is up to the orthodontist to discern the reliable scientific knowledge from those who have errors or bias - acquiring for their clinical practice what will, for example, reduce error rates, waste, unsuccessful therapies and unnecessary exams.^1^ ^,^ ^2^

Evidence-based Orthodontics can become a challenge for clinicians. This is because published papers often present information that makes understanding scientific knowledge a complex task.^3^ ^,^ ^4^ A substantial level of experience in statistical understanding is necessary in the critical reading of the research, the methodology used, data analysis and interpretation of the results, for the acquisition of conclusions that will reduce the uncertainties in decision making, in view of the variability of available options.^2^ ^,^ ^5^ ^-^ ^7^

Statistics are known to have a direct connection to mathematics. And the culture of fear and anxiety that surrounds it makes the assimilation of statistical concepts and methods complex.^8^ Some studies show that graduate students, despite understanding the importance of biostatistics, do not have the skills to apply it correctly in scientific research; and that attitudes, successes and failures in face of statistical challenges are linked to basic knowledge.^6^ ^,^ ^9^ ^-^ ^11^ This ends up having an impact on scientific publications. Studies showed that it is common to find errors such as incompatible study design, inadequate analysis and inconsistent interpretations.^12^ ^-^ ^14^

The basic concepts, which are fundamental to avoid errors, are often easy to forget, impacting the choice of statistical tests used in the data analysis. In addition, most statistical software does not guide the user in choosing the most appropriate statistical test for the research, generating scientific publications that do not contribute to the solution of a clinical problem, due to the wrong data analysis.^15^

Therefore, the objective of the present article is to clearly review some essential concepts of biostatistics that will assist clinical orthodontists in understanding scientific research for an evidence-based clinical practice, in addition to indicate the main errors observed in published articles. Then, it will be presented the updated version of a PowerPoint^®^ guide, originally published in 2010, to assist in choosing the appropriate statistical test.^16^ This guide is useful for readers, authors and reviewers of scientific articles.

## BASIC CONCEPTS

Biostatistics is a method used to describe or analyze data obtained from a sample that represents a population. It is used in studies in which variables are related to living beings.^17^ ^,^ ^18^

### WHAT IS A VARIABLE AND HOW IS IT MEASURED?

Variable is a characteristic or condition that can be measured or observed in the sample or population. It can assume different values from one sampling unit to another or in the same unit over time. It is important to know how to classify the variable according to the data it generates. To understand the classifications of the variables regarding the scale used and the type of participation in the study, see Tables 1 and 2, respectively.^8^ ^,^ ^17^ ^,^ ^18^

**Table 1:** Classification of the types of variables according to the scale used.

	NUMERIC OR QUANTITATIVE VARIABLES It is expressed in numbers		NON-NUMERIC, CATEGORICAL OR QUALITATIVE VARIABLE It is expressed in words	
	Discrete	Continuous	Ordinal	Nominal
Concept	It only assumes integer values such as 0, 1, 2, 3, 4 and so on, not allowing fractional values. It is related to counts.	Assumes numeric values both integer and fractional (decimal). It is related to the measurement of quantities.	Represents two or more categories in which the data has ordering or hierarchy.	It represents two or more categories in which there is no order or hierarchy.
Examples	DMFT index; number of erupted teeth.	Cephalometrics measurements; Anterior open bite, in millimeters; Treatment time, in months.	Education level; Pain intensity (absent, low, moderate, severe); Plaque index.	Gender: female or male; Blood type: A, B, AB or O; Angle classification: I, II or III; Questions where the answers can be “yes” or “no”.

**Table 2:** Classification of the types of variables according to the type of participation in the study.

	DEPENDENT VARIABLE Called “Response Variable”	INDEPENDENT VARIABLE Also known as “Explanatory” or “Predictor”
Concept	It is the event or characteristic that you want to discover or explain. It represents a quantity whose appearance, disappearance, increase, decrease, etc. depends on how the independent variable is handled by the researcher.	It is the determining factor, condition or cause that makes it possible to predict a response, effect or consequence. It can vary during the study or be controlled, but is not affected by any other variable within the experiment.
Example	In one study, it is intended to ascertain the need for orthodontic treatment based on gender, age, education, socioeconomic level and perception of oral health. Thus, the response variable (dependent) of the study is the “need for orthodontic treatment”, while the others are explanatory (independent) variables.	

### THE IMPORTANCE OF NORMAL DISTRIBUTION

**Figure 1:**
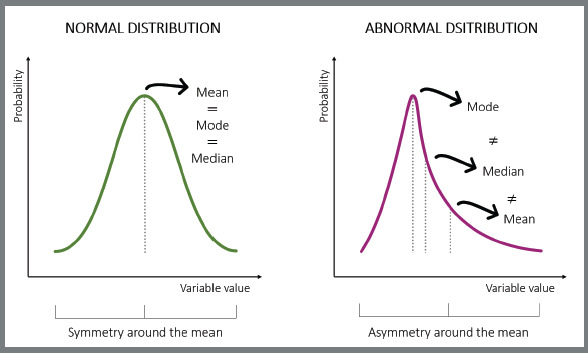
Graphical exemplification of normal and abnormal distributions.

A distribution in biostatistics refers to a mathematical model that relates values of a variable and the probability of occurrence of each value. It should be clarified that whenever there is a quantitative variable that will be analyzed, it is assumed to verify the normality of the data distribution, by statistical test and/or histogram, according to the need. Some statistical tests require a distribution with normal characteristics as a requisite. Where the data is concentrated around the average and from there they are dispersed in a symmetrical way, with a characteristic bell-shaped graph. When the distribution is different from the normal, preference should be given to the use of median and interquartile deviation.^17^ ^-^ ^19^ The graphical elucidation of normal and abnormal data distributions can be seen in Figure 1.

### HOW SHOULD THE DATA BE PRESENTED? (DESCRIPTIVE STATISTICS)

The organization and presentation of these data, made by appropriate methods, can be summarized, known as descriptive statistics. This concept is the initial step for an appropriate selection and use of statistical tests. Descriptive statistics can be divided into frequencies and/or summary measures of central tendency and dispersion (Table 3).^8^ ^,^ ^17^ ^,^ ^18^

**Table 3:** Ways of organizing the data in the descriptive analysis.

	FREQUENCY DISTRIBUTION Used for both numeric (quantitative) and non-numeric (qualitative) data. Count of occurrence in the sample by frequency, rate or ratio		SUMMARY MEASURES The summary provides the distribution of data, by central tendency or by variability	
	FREQUENCY	RATE OR RATIO	CENTRAL TENDENCY	VARIABILITY
C O N C E P T	Absolute frequency: Represents the number of times that each category appeared in the sample. It is the result of counting the number of sample units belonging to each category. Relative frequency: Assumes numeric values both integer and fractional (decimal). It is related to the measurement of quantities. It is the division between the absolute frequency of a category and the total frequencies observed. Percent frequency: It is the relative frequency multiplied by 100%.	Rate: It is the relative frequency multiplied by 1000, 10000 or 100000. Ratio: It is the relative frequency of one category divided by the relative frequency of another category.	Mean: Is the quotient between the sum of the data and the total number of observations (n). It should be used in quantitative data with normal distribution. Median: Represents who is in the middle of the ranked sample. It can be used in non-normal quantitative or ordinal data. When normal distribution is achieved, its value is similar to that of the mean. Mode: Represents the most often value. It can be used in any type of variable, but it is of little use in publishing scientific studies	Also known as “dispersion measures”, as they reveal how the data varies or is distributed around its midpoint. **Amplitude:** Is the difference between the highest and lowest value in a data set. **Standard deviation (SD):** Is the value that represents the symmetric average dispersion of data around the mean of a data set. It is used in quantitative data with normal distribution. **Variance:** Is the standard deviation value raised to the square. **Variation coefficient:** Is the relative dispersion of the data, represented by the ratio between the standard deviation and the mean, multiplied by 100. **Percentile:** Percentiles are the 99 values that separate a series into 100 equal parts. **Quartile:** Quartile are the values of a series that divide it into four equal parts. First quartile (Q1) includes the first 25% of the data, second quartile or median (Q2) includes the first 50% of the data, third quartile includes the first 75% of the data (Q3). **Interquartile deviation:** Difference between the Q3 (P75) and Q1 (P25), which is not influenced by extreme values. Should be used when non-normal data are being evaluated.

### WHY USE STATISTICAL TESTS? (INFERENTIAL STATISTICS)

Inferential statistics allow comparing samples or predicting behaviors of variables. This tool establishes conclusions based on a small portion of a population, with a minimum and previously determined margin of error. Statistical tests are used to quantify the uncertainty of decision making by means of probabilistic principles.^6^ ^,^ ^17^

Allows the researcher to have a degree of reliability in the statements assumed in the sample, regarding the population. Thus, when the reader realizes that the published study performed statistical tests, he must ask: *“How likely am I to trust these results?”* or *“How much uncertainty is there in the results for an extrapolation of the results (generalization)?".* These questions should be asked at the beginning of the study, in order to define the chances of error, the confidence and the estimated margins of the population parameter of your sample. The following are the concepts of interest:^6^ ^,^ ^17^ ^-^ ^19^ ^,^ ^20^

1. Significance, or α level (*p*-value): ): it represents the chance that the researcher is wrong in stating that there is a difference (or significance), and the difference, in fact, does not exist. Known as type I error or false positive, it can be predetermined at the beginning of the study as 1% or 5%. It can be said that when you have a *p-*value less than the level of significance, then you have a real difference between samples or groups, when applied in groups comparison tests.
2. *β* error: known as a type II error, or false negative, it represents the chance of the researcher making a mistake in stating that there is no difference when the difference is true. A maximum value of 20% is allowed.
3. Study power (1 - *β*) : represents the chance for the researcher to be sure that there is a difference when it really exists. It is also defined by the researcher before data collection begins, and is usually at least 80%, or 0.8 (1 - 0.2).
4. Confidence interval: represents the estimate of a sample parameter for a population parameter. Contains upper and lower limits, defined according to the stipulated level of significance. For a 95% confidence interval, we have that for every 100 studies performed, within the same methodology and (n), but with different subjects from the same population, it must be estimated that the population parameter is present in the data distribution of 95 studies. However, there is no need to carry out the 100 studies for this estimate. Just perform a single study and define this interval by 95% CI = mean ± (1.96 x standard error). Therefore, standard error = standard deviation divided by √n .

Currently, journals and reviewers have requested in the results not only the *p*-value, but also the referring confidence interval (CI). Some years ago, only a few studies with with multivariate analyzes reported the CI found.^21^ A systematic review^22^ showed that the interpretation of the CI is important, but it rarely occurs in those randomized clinical trials where the effects of treatments were not statistically significant. This can lead to the abandonment of future research or to a clinical practice based on invalid conclusions.

### TYPES OF STUDIES

The execution of a study must always be planned, and this plan for conducting the research is called research design. It must follow specific standards and techniques, according to the nature of the study.^6^ ^,^ ^17^ ^-^ ^19^

**Figure 2:**
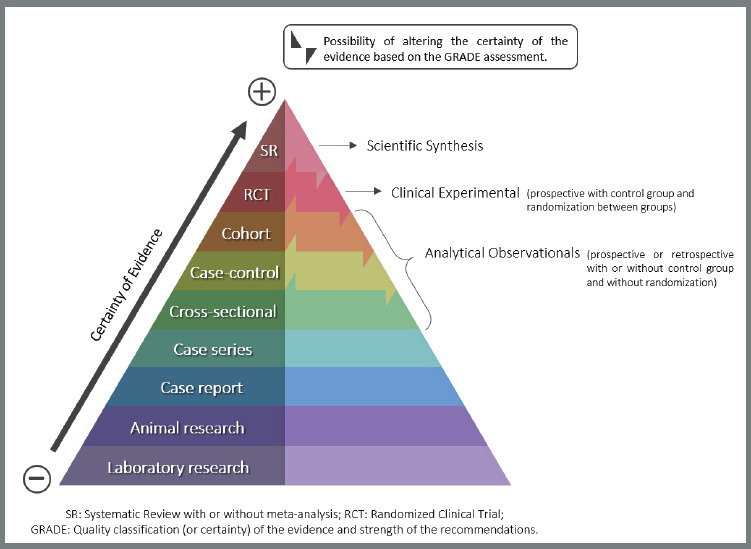
Pyramid of evidence and the types of studies included.

The quality of research designs is related to the strength of recommendation and applicability to the patient.^18^ This difference between the degree of strength of the types of studies can be seen in Figure 2, representing a pyramid of evidence.

This pyramid incorporates the suggestion of Murad et al.^23^ that considers not only the study design, but also the assessment of the certainty of the evidence, examined by the GRADE tool (Grading of Recommendations Assessment, Development and Evaluation).^24^ So that, for example, a cross-sectional study very well performed can produce a much better quality of results than a case-control study not so well developed, therefore, producing a greater impact on clinical decision. This type of change in the strength of the recommendation can occur in studies present from the middle to the top of the pyramid, and will be seen below:^8^ ^,^ ^18^ ^,^ ^19^

1. Analytical observational studies: They are studies in which the objective is the observation, description and analysis of variables by means of the comparison of groups. There is no randomization process in the selection of participants, and the exposure was not assigned by the researcher. They are subdivided into three categories, as described bellow.
  » Cross-sectional: It is considered a “portrait study”. It determines the situation of interest and outcome in a single moment, assessing the prevalence and relationship between variables, comparing exposed and unexposed or with disease and without disease. Example: to analyze the association between gingival inflammation (present/absent) and the use of orthodontic appliance (exposure) in a single moment of treatment, comparing with patients without orthodontic treatment (control).
  » Cohort: It is a longitudinal study, considered a “film study”. It starts from the exposure to the outcome (disease), and observes over time individuals exposed and not exposed (control group) to a factor - who have not yet developed the outcome of interest -, assessing incidence, carrying out supervised monitoring and establishing etiology and risk factors. Although it is generally prospective, when the data registration coincides with the beginning of the research; there are retrospective cohorts, when the research is initiated after recording the data. Example: analyzing the development of gingival inflammation (incidence) during orthodontic treatment (exposure), with evaluation of gingival condition at the beginning and end of treatment, compared to non-orthodontic patients.
  » Case-control: It is also a longitudinal study and evaluates individuals who already have the disease of interest, comparing them with a control group (individuals without the disease), measuring the exposures or interventions performed during the study. Although it is generally retrospective, they can be carried out prospectively. Example: comparative analysis of orthodontic patients with and without gingival inflammation (disease, or outcome) among patients who did or did not use daily mouthwash. In this case, starts from the disease to the exposure.
2. Randomized Clinical Trial: This is a simulation study of the reality in which an exposure or intervention in the experimental sample occurs, in comparison to a control group. The main feature is the allocation of research subjects being carried out by randomization between groups. It is a highly controlled study. However, the randomization method can fail, especially when small samples are analyzed.
3. Synthesis: This category includes the secondary study called “Systematic Review”. It uses primary studies as a source of data to obtain the answer to a key question. It is a scientific investigation carried out under a rigorous methodology for both data searches and analyzes, and the consequent determination of the certainty of the available evidence. When possible, a “meta-analysis” is carried out, which is the statistical analysis to combine the results of the included primary studies. It is at the top of the evidence pyramid for clinical decision-making (Fig 2).

### MAIN ERRORS IN THE STATISTICAL METHODOLOGY OBSERVED IN THE PUBLISHED ARTICLES

Articles that will be submitted to journals must be very well written and designed. This requires that the study be conducted in a reliable manner, allowing the correct description of all the steps performed and the consequent ease of reading and acceptance of the article by the reviewers^25^. Below are the most common errors found in published articles, regarding the statistical methodology employed.

### USE OF COLUMN / BAR GRAPH FOR QUANTITATIVE VARIABLES

**Figure 3:**
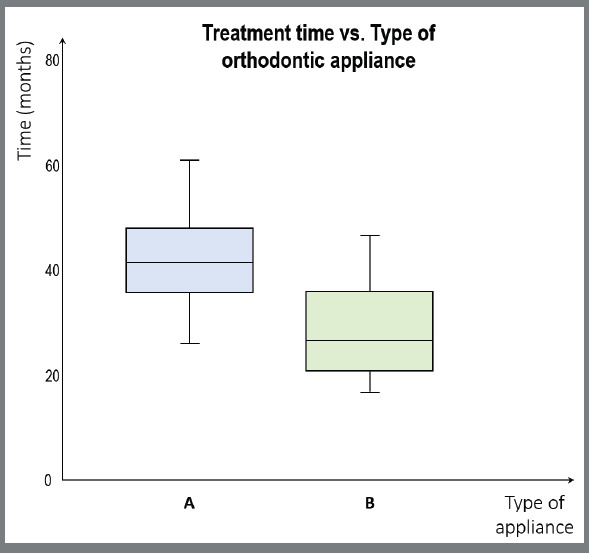
Exemplification of a box-plot type graph of a hypothetical study in which it was sought to assess whether there was a difference in treatment time between two types of orthodontic appliances.

Column graphics should be used for frequency graphics, as each column represents a category. When we have numeric variables, we should use the box-plot graph (Fig 3) for independent samples and the line graph for data over time^8^ ^,^ ^18^. The box-plot, unlike the column graphic, allows us to observe the summary measure (mean or median) and the dispersion of the obtained values.

### USE OF PARAMETRIC TESTS WHEN NORMALITY IS NOT ACHIEVED

Parametric tests are more powerful than non-parametric tests, but they presume a normal distribution of data. Numerical data with abnormal distribution should be analyzed as if they were qualitative data. The use of a parametric test in this situation implies greater ease in rejecting the null hypothesis, which may not represent the population’s reality.^8^ ^,^ ^19^

### USE OF MEAN AND STANDARD DEVIATION WHEN THERE IS AN ABNORMAL DISTRIBUTION

Although some researchers correctly use non-parametric tests when the assumption of normality is broken, they sometimes incorrectly use the mean and standard deviation when presenting data. These should not be used exactly due to the asymmetric distribution. In this case, use the nonparametric reference that divides the data in half: the median and its deviation (interquartile deviation).^1^ ^,^ ^8^ ^,^ ^17^

### OVERSIZED OR UNDERSIZED SAMPLES

Every sample has a minimum number of sample units needed to represent the population. When you have a sample below that quantity (undersized), only large differences can be detected in a significant way. In addition, small samples tend to have an abnormal distribution, which would lead to the use of less robust tests; while large samples are not practical, since they increase the cost and time required for the study.^17^ ^,^ ^18^

### A GUIDE TO ASSIST IN CHOOSING THE APPROPRIATE STATISTICAL TEST

In order to obtain a reliable statistical result that allows extrapolation to the population of interest, it is extremely important to know which test is the most suitable for the study. A PowerPoint^®^ guide to assist in choosing the statistical test was published in 2010^16^, and since then it has been widely used by countless researchers. With more than 244 thousand accesses (02/10/2021), this is the article with the highest number of downloads in the SciELO collection for the dentistry area. The version 2020 3.0 presents a new layout and has additional multivariate analyzes. In addition, this version provides the paths for running the tests on several free software, such as Jamovi (version 1.2, Sydney, Australia) and BioEstat (version 5.3, Amazonas, Brazil), as well as on the website www.vassarstats.net (VassarStats, Richard Lowry - United States). The download can be done according to the preferred language (Portuguese, English or Spanish), through the following link:

http://www.ppgo.propesp.ufpa.br/index.php/br/programa/noticias/todas/176-tutorial-teste-estatistico-para-pesquisa-cientifica

**Figure 4:**
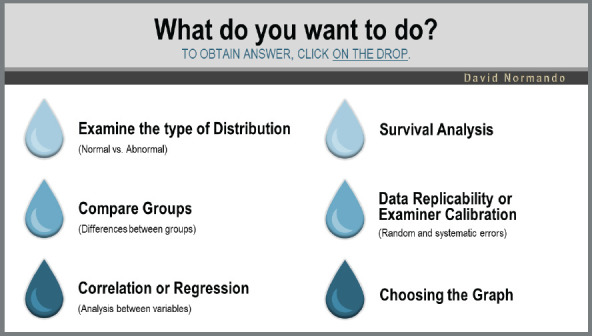
Initial menu of the tutorial.

The use is simple and must be done in the “presentation mode”. The INITIAL MENU represents the objective intended by the researcher. There are six possible options (Fig 4), where it is possible to arrive at the desired answer through a sequence of clicks on drops.

By clicking on the option “Examine the type of distribution”, you have the direct answer of which test you can perform to examine the distribution of data for a quantitative variable. The same occurs when clicking on the drop “Survival analysis”, in which the possible options for survival tests are directly available.

In the other options of the initial menu, there is a hyperlink to the submenus:

*» Comparison menu:* allows obtaining the specific statistical test for the difference between two or more paired or independent samples with or without normal distribution.
*» Correlation menu:* indicates the analyzes for correlation and/or modeling between two or more variables.
*» Replicability menu:* indicates the measurement accuracy tests for the analysis of random and systematic errors.
*» Graphic menu:* provides the researcher with the appropriate graph for the type of sample and objective of the study.

**Figure 5:**
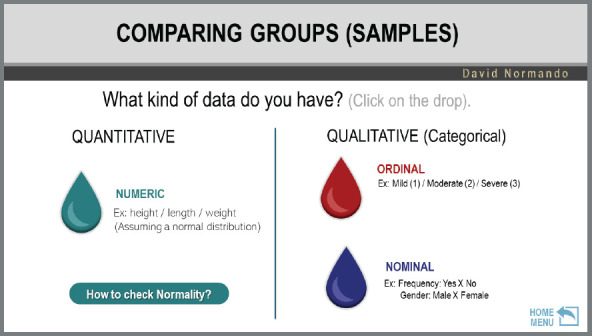
Example of a tutorial submenu.

So that, after a sequence of clicks on drops, it is possible to obtain the desired response. Figure 5 exemplifies a submenu - in this case, the comparison.

**Figure 6:**
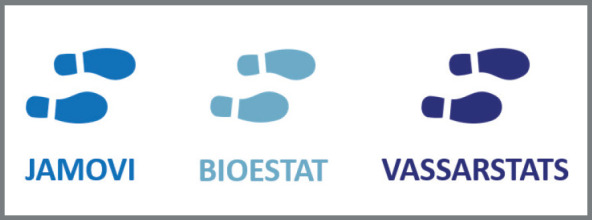
Icons that represent the execution path of the Jamovi, Bioestat, and Vassarstats software.

This sequence of clicks requires basic knowledge about types of variables (Table 1) and data distribution (Fig 1). It is also necessary to understand the difference between dependent (paired) and independent (unpaired) samples. Paired samples are those in which the comparison is dependent on the same individual (before ***vs.*** after; right ***vs.*** left; T1 ***vs.*** T2 ***vs.*** T3). Independent samples are those in which there is a comparison between different individuals. After identifying the desired answer, you can obtain the test execution path in both BioEstat (not available in the English version) and Jamovi, and, in some cases, in VassarStats, by clicking on the corresponding icon that will appear (Fig 6).

In this update, when you click on an item by mistake, it is possible to return to the submenu in which it was already found, providing greater agility during use.

## CONCLUSION

It is important that readers and reviewers of journals can identify when a research uses inadequate statistical analysis, disregarding fundamental concepts. The new version of the "Tutorial for choosing the test" presented is a path to the most appropriate use of statistical tests, allowing the correction of wrong choices.

## References

[1] An TL, Cuochi OA (2004). Utilization of statistics in orthodontia. Rev Dent Press Ortod Ortop Facial.

[2] Silva GAR (2013). The decision-making process in clinical practice medicine as a state of the art. Rev Soc Bras Clin Med.

[3] Rumsey D, Rumsey D (2010). Most used hypothesis tests. Statistics for dummies.

[4] Schneider LR, Pereira RPG, Ferraz L (2018). Evidence-based practice in the context of primary health care. Saúde Debate.

[5] Mariano MTS, Januzzi E, Grossmann E (2009). Scientific evidence-based orthodontics incorporating science into clinical practice. Rev Dent Press Ortod Ortop Facial.

[6] Polychronopoulou A, Eliades T, Taoufik K, Papadopoulos MA, Athanasiou AE (2011). Knowledge of European orthodontic postgraduate students on biostatistics. Eur J Orthod.

[7] Rodrigues CF, Lima FJC, Barbosa FT (2017). Importance of the proper use of basic statistics in clinical research. Rev Bras Anestesiol.

[8] Hernandez JAE, Santos GR, Silva JO, Mendes SLL, Ramos VCB (2015). Evidence of validity of the anxiety scale in statistics in psychology students. Psychol Science Prof.

[9] El Tantawi MM (2009). Factors affecting postgraduate dental students' performance in a biostatistics and research design course. J Dent Educ.

[10] Murthykumar K, Veeraiyan DN, Prasad P (2015). Impact of video based learning on the performance of postgraduate students in biostatistics a retrospective study. J Clin Diagn Res.

[11] Chowdhury SK (2018). Prior knowledge, sex and students' attitude towards statistics a study on postgraduate education students. Am J Educ Res.

[12] Lucena C, Lopez JM, Pulgar R, Abalos C, Valderrama MJ (2012). Potential errors and misuse of statistics in studies on leakage in endodontics. Int Endod J.

[13] Gates S, Ealing E (2018). Reporting and interpretation of results from clinical trials that did not claim a treatment difference survey of four general medical journals. BMJ Open.

[14] Velez FGS, Pinto CB, Chalita M, Falcao D, Fregni F, Amorim R (2016). Avoiding mistakes while writing scientific manuscripts in medical and health sciences. Geriatr Gerontol Aging.

[15] Mann PS (2006). Introduction to statistics.

[16] Normando D, Tjaderhane L, Quintão CCA (2010). A powerpoint-based guide to assist in choosing the suitable statistical test. Dental Press J Orthod.

[17] Padovani CR (2012). Biostatistics.

[18] Estrela C (2005). Scientific methodology.

[19] Demathé A, Silva ARS, De Carli JP, Goiato M, Miyahara G (2012). Evidence-based dentistry optimizing practice and research. RFO UPF.

[20] Zientek LR, Yetkiner OZE, Ozel S, Allen J (2012). Reporting confidence intervals and effect sizes collecting the evidence. Career & Technical Education Research.

[21] Polychronopoulou A, Pandis N, Eliades T (2011). Appropriateness of reporting statistical results in orthodontics the dominance of P values over confidence intervals. Eur J Orthod.

[22] Gewandter JS, McDermott MP, Kitt RA, Chaudari J, Koch JG, Evans SR (2017). Interpretation of CIs in clinical trials with non-significant results systematic review and recommendations. BMJ Open.

[23] Murad MH, Asi N, Alsawas M, Alahdab F (2016). New evidence pyramid. Evid Based Med.

[24] Atkins D, Eccles M, Flottorp SA, Guyatt GH, Henry D, Hill S (2004). Systems for grading the quality of evidence and the strength of recommendations I critical appraisal of existing approaches The GRADE Working Group. BMC Health Serv Res.

[25] Faber J (2017). Writing scientific manuscripts most common mistakes. Dental Press J Orthod.

